# Supplementary material for: Tales of 1,008 small molecules: phenomic profiling through live-cell imaging in a panel of reporter cell lines
Source: Sci Rep. 2020 Aug 6;10:13262. doi: 10.1038/s41598-020-69354-8 (PMC7411054; doi:10.1038/s41598-020-69354-8)
Supplement: Supplementary file 2 — Supplementary Information 2. [file 41598_2020_69354_MOESM2_ESM.pdf]

Tales of 1,008 Small Molecules: Phenomic Profiling through Live-cell Imaging in a Panel of Reporter Cell Lines

Michael J. Cox[#,1], Steffen Jaensch[#,1,*], Jelle Van de Waeter[1], Laure Cougnaud[2], Daan Seynaeve[2], Soulaiman Benalla[1], Seong Joo Koo[1], Ilse Van Den Wyngaert[1], Jean-Marc Neefs[1], Dmitry Malkov[3], Mart Bittremieux[1], Margino Steemans[1], Pieter J. Peeters[1], Jörg Kurt Wegner[1], Hugo Ceulemans[1], Emmanuel Gustin[1], Yolanda T. Chong[1,4], and Hinrich W.H. Göhlmann[1]

[#] Co-first authors

[1] Janssen Pharmaceutica N.V., Beerse, Belgium

[2] Open Analytics N.V., Antwerpen, Belgium

[3] MilliporeSigma, Saint Louis, MO, USA

[4] Present address: Recursion, Salt Lake City, UT, USA

* Corresponding author: Steffen Jaensch, Janssen Pharmaceutica N.V., Turnhoutseweg 30, 2340 Beerse, Belgium, SJAENSCH@its.jnj.com

Supplementary Figure S6

t-SNE maps for each of the 15 cell lines, analogously to Fig. 3 in the main manuscript. This figure was created in R version 3.6.1 (https://www.R-project.org/) using the ggplot2 package version 3.2.1 (https://ggplot2.tidyverse.org/).

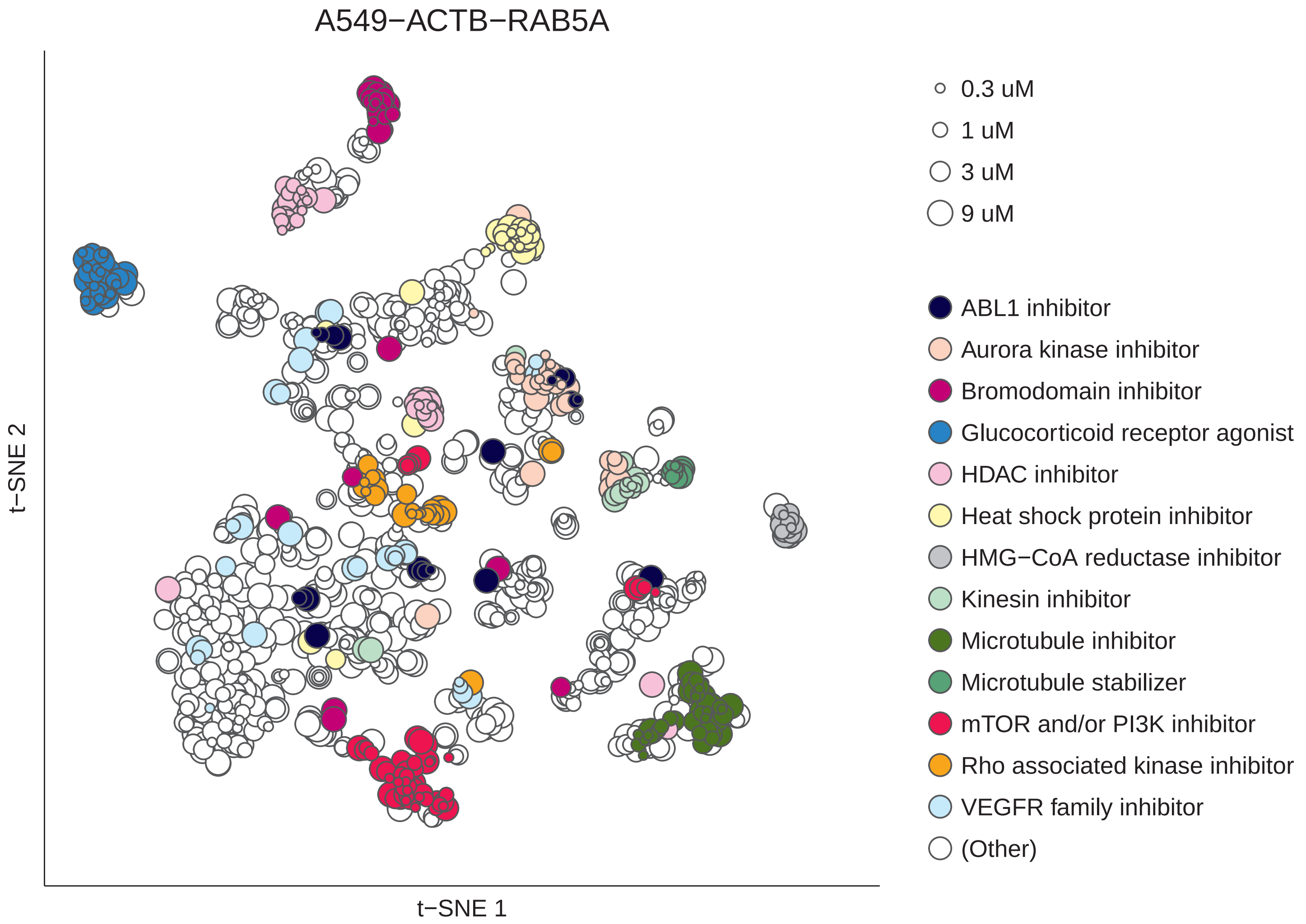
A549−ACTB−RAB5A
t−SNE 2
t−SNE 1
0.3 uM
1 uM
3 uM
9 uM
ABL1 inhibitor
Aurora kinase inhibitor
Bromodomain inhibitor
Glucocorticoid receptor agonist
HDAC inhibitor
Heat shock protein inhibitor
HMG−CoA reductase inhibitor
Kinesin inhibitor
Microtubule inhibitor
Microtubule stabilizer
mTOR and/or PI3K inhibitor
Rho associated kinase inhibitor
VEGFR family inhibitor
(Other)

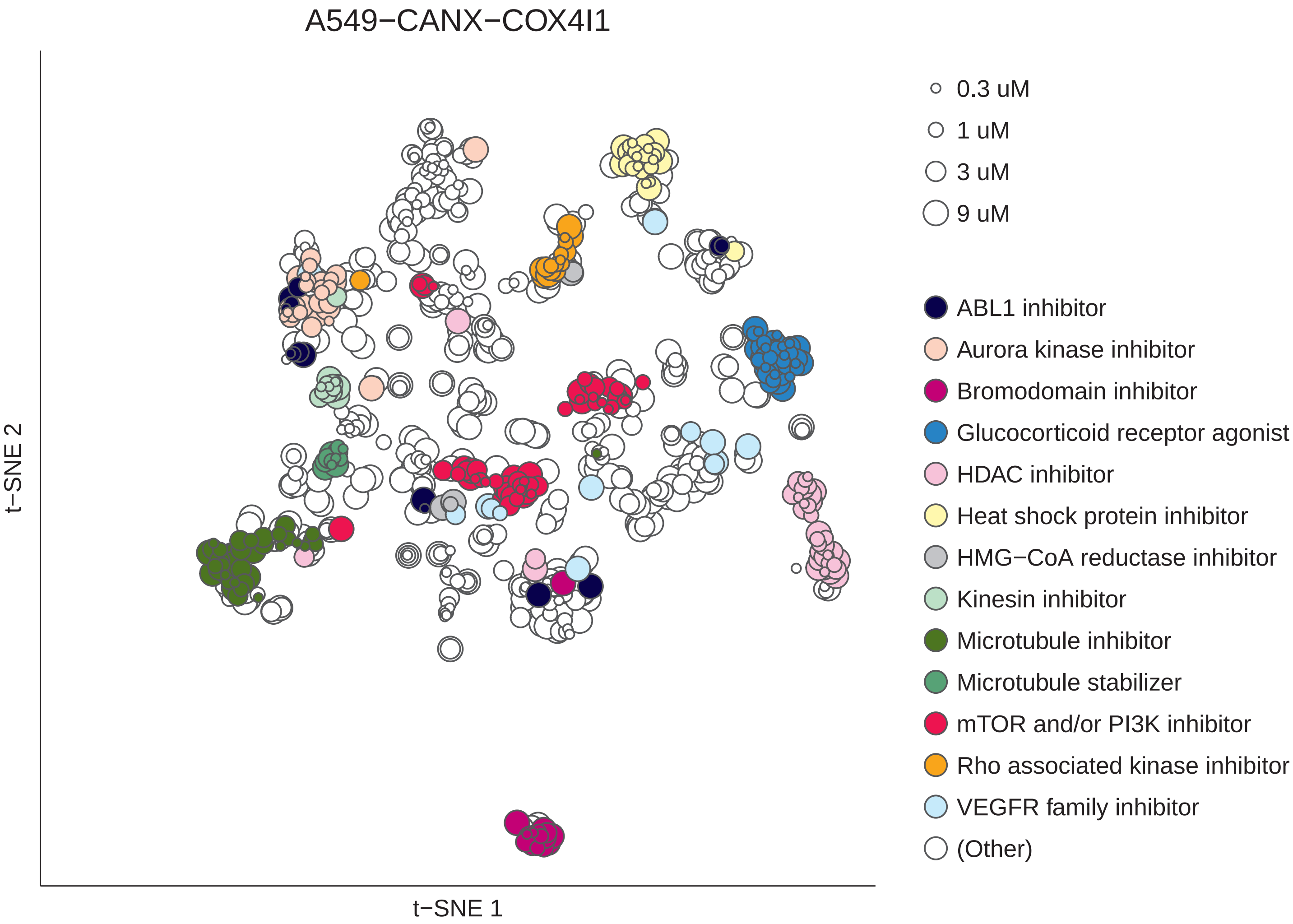

A549−CANX−COX4I1
t−SNE 2
t−SNE 1
0.3 uM
1 uM
3 uM
9 uM
ABL1 inhibitor
Aurora kinase inhibitor
Bromodomain inhibitor
Glucocorticoid receptor agonist
HDAC inhibitor
Heat shock protein inhibitor
HMG−CoA reductase inhibitor
Kinesin inhibitor
Microtubule inhibitor
Microtubule stabilizer
mTOR and/or PI3K inhibitor
Rho associated kinase inhibitor
VEGFR family inhibitor
(Other)

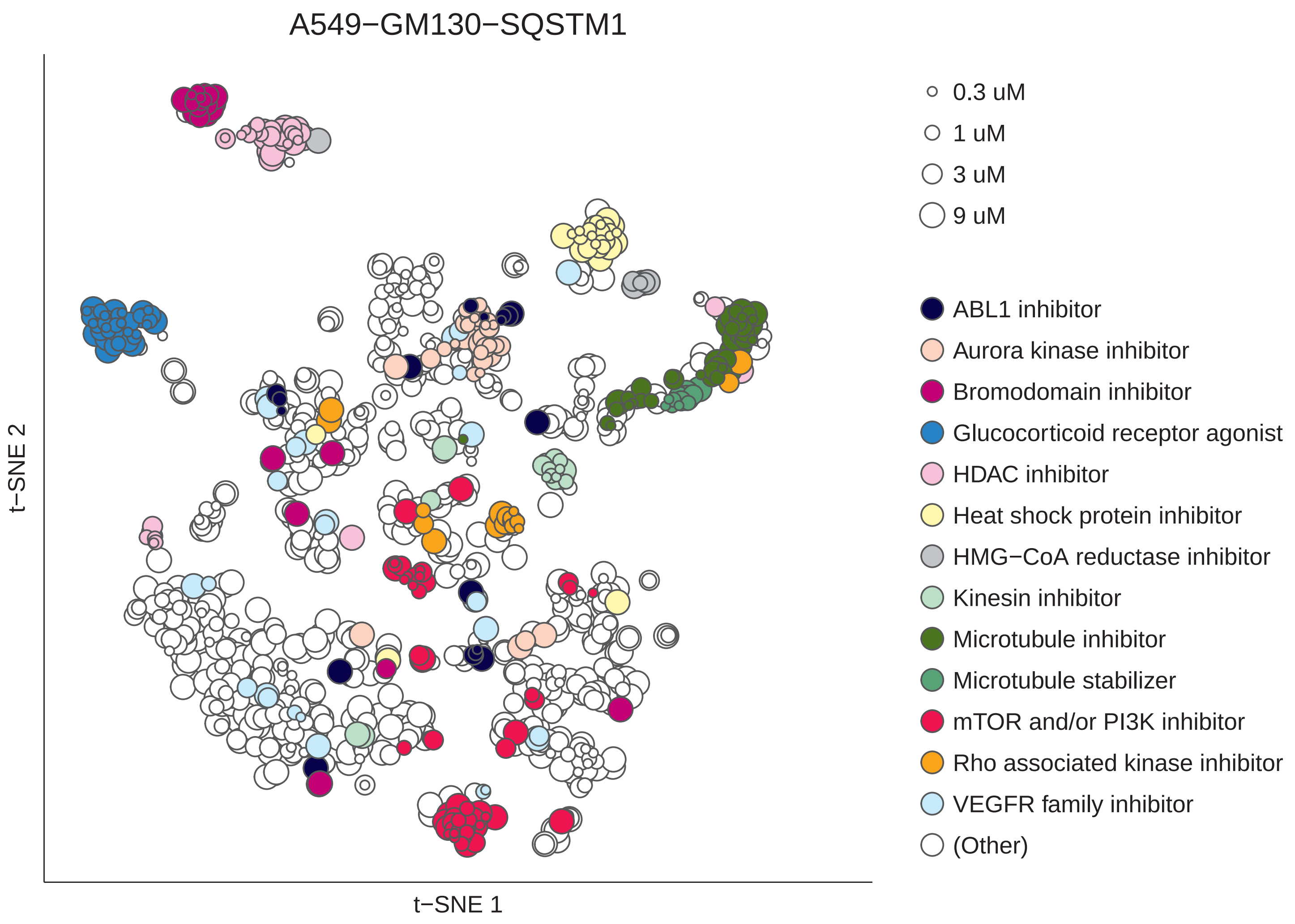
A549−GM130−SQSTM1
t−SNE 2
t−SNE 1
0.3 uM
1 uM
3 uM
9 uM
ABL1 inhibitor
Aurora kinase inhibitor
Bromodomain inhibitor
Glucocorticoid receptor agonist
HDAC inhibitor
Heat shock protein inhibitor
HMG−CoA reductase inhibitor
Kinesin inhibitor
Microtubule inhibitor
Microtubule stabilizer
mTOR and/or PI3K inhibitor
Rho associated kinase inhibitor
VEGFR family inhibitor
(Other)

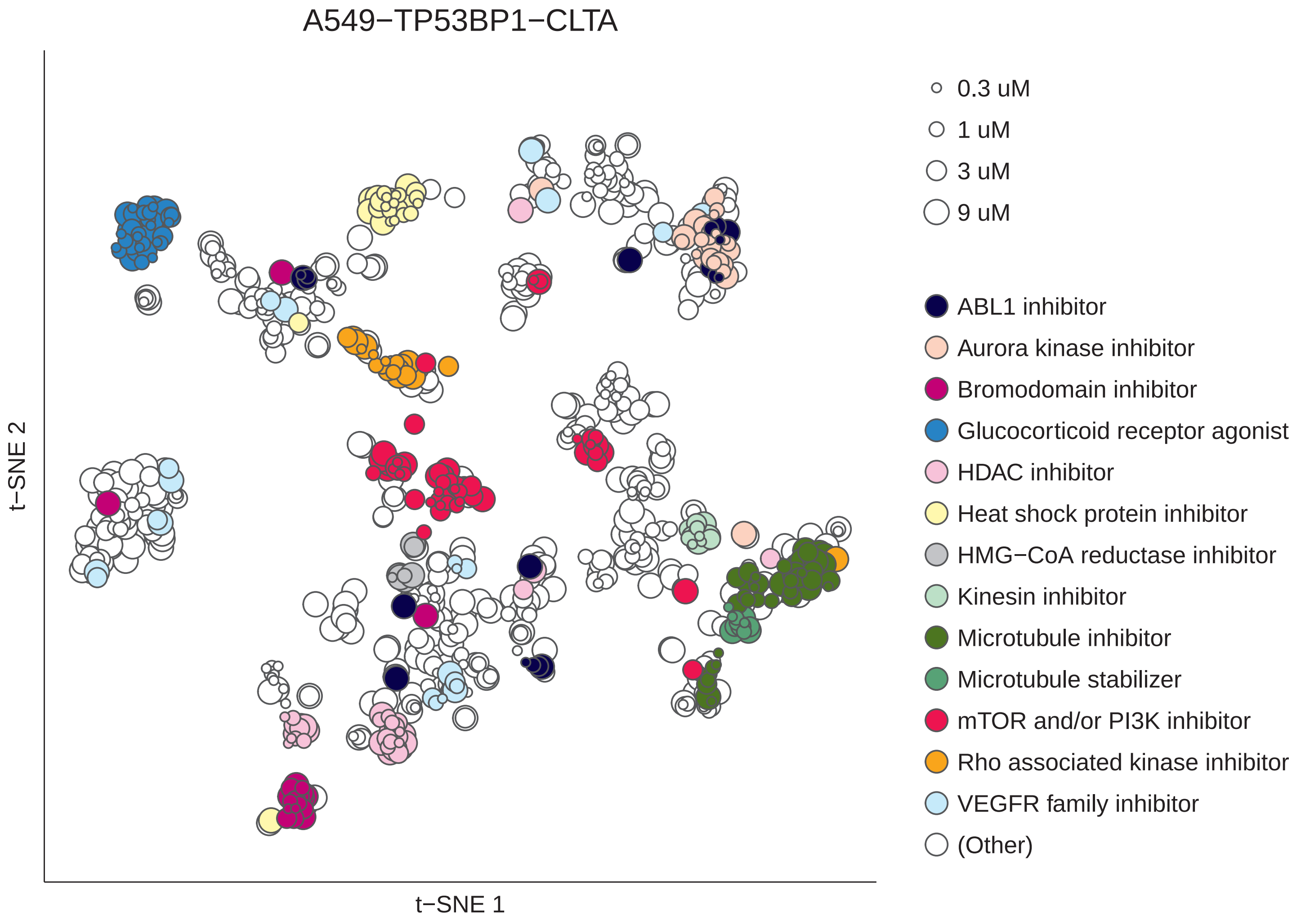
A549−TP53BP1−CLTA
t−SNE 2
t−SNE 1
0.3 uM
1 uM
3 uM
9 uM
ABL1 inhibitor
Aurora kinase inhibitor
Bromodomain inhibitor
Glucocorticoid receptor agonist
HDAC inhibitor
Heat shock protein inhibitor
HMG−CoA reductase inhibitor
Kinesin inhibitor
Microtubule inhibitor
Microtubule stabilizer
mTOR and/or PI3K inhibitor
Rho associated kinase inhibitor
VEGFR family inhibitor
(Other)

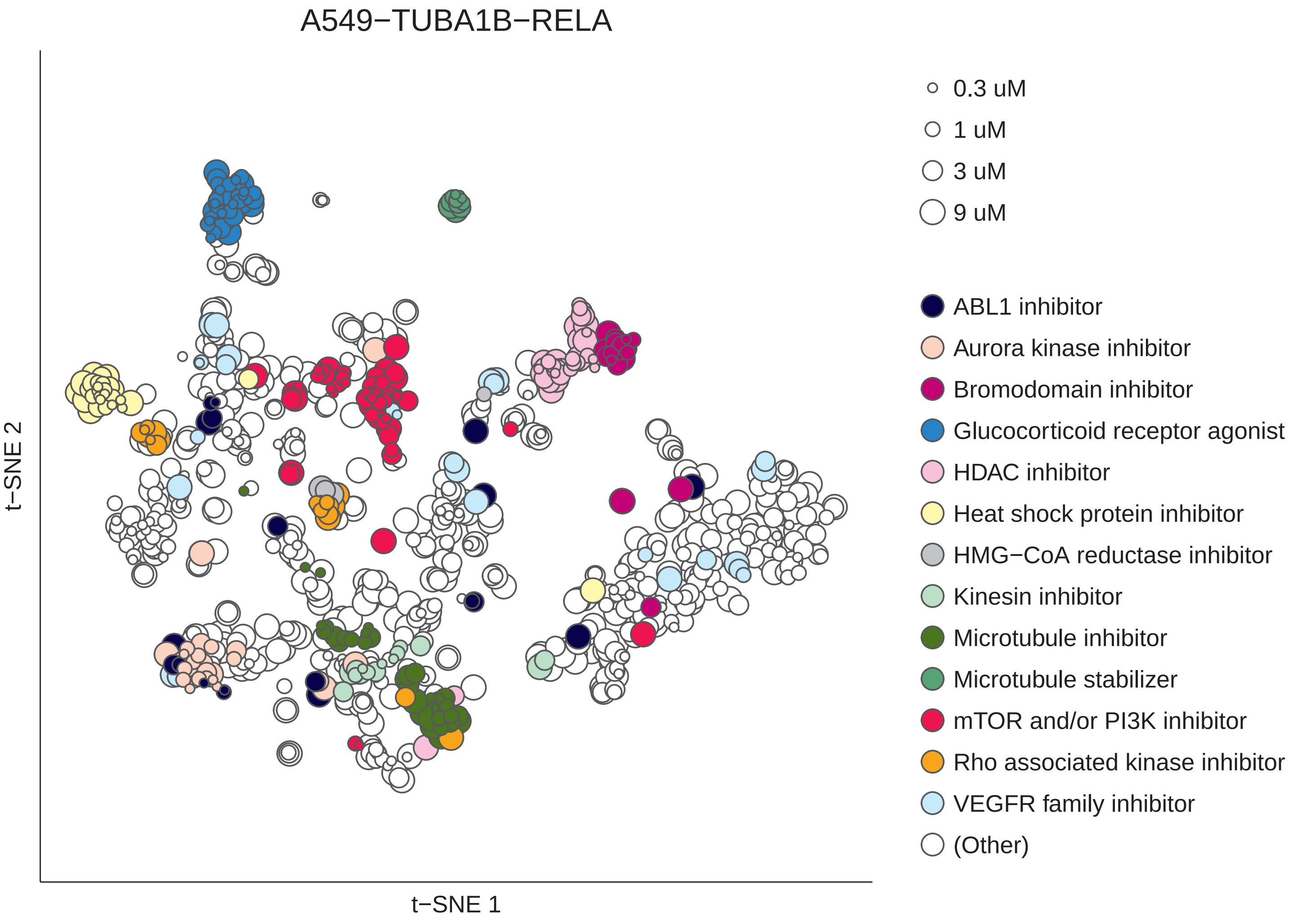
A549−TUBA1B−RELA
t−SNE 2
t−SNE 1
0.3 uM
1 uM
3 uM
9 uM
ABL1 inhibitor
Aurora kinase inhibitor
Bromodomain inhibitor
Glucocorticoid receptor agonist
HDAC inhibitor
Heat shock protein inhibitor
HMG−CoA reductase inhibitor
Kinesin inhibitor
Microtubule inhibitor
Microtubule stabilizer
mTOR and/or PI3K inhibitor
Rho associated kinase inhibitor
VEGFR family inhibitor
(Other)

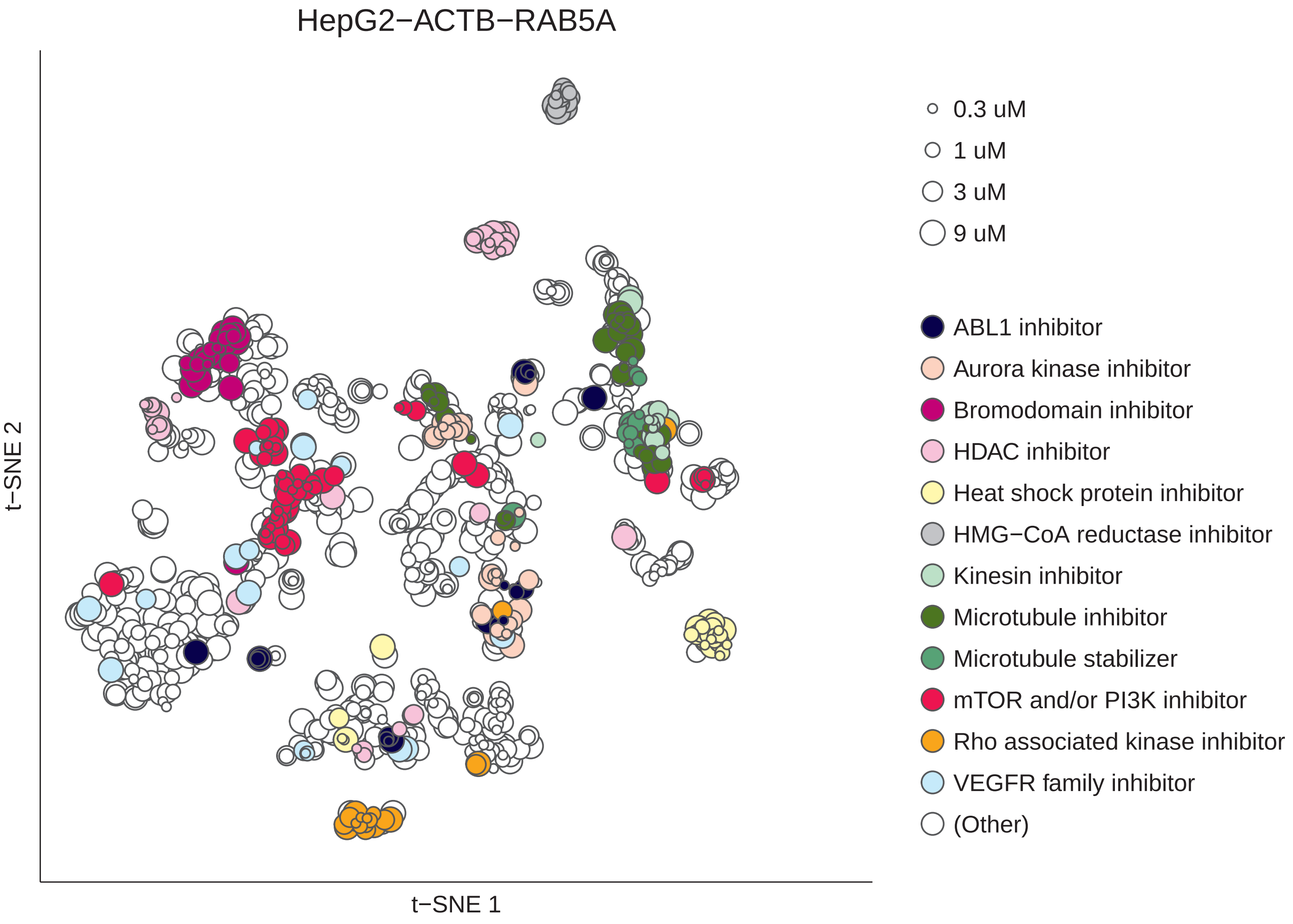
HepG2−ACTB−RAB5A
t−SNE 2
t−SNE 1
0.3 uM
1 uM
3 uM
9 uM
ABL1 inhibitor
Aurora kinase inhibitor
Bromodomain inhibitor
HDAC inhibitor
Heat shock protein inhibitor
HMG−CoA reductase inhibitor
Kinesin inhibitor
Microtubule inhibitor
Microtubule stabilizer
mTOR and/or PI3K inhibitor
Rho associated kinase inhibitor
VEGFR family inhibitor
(Other)

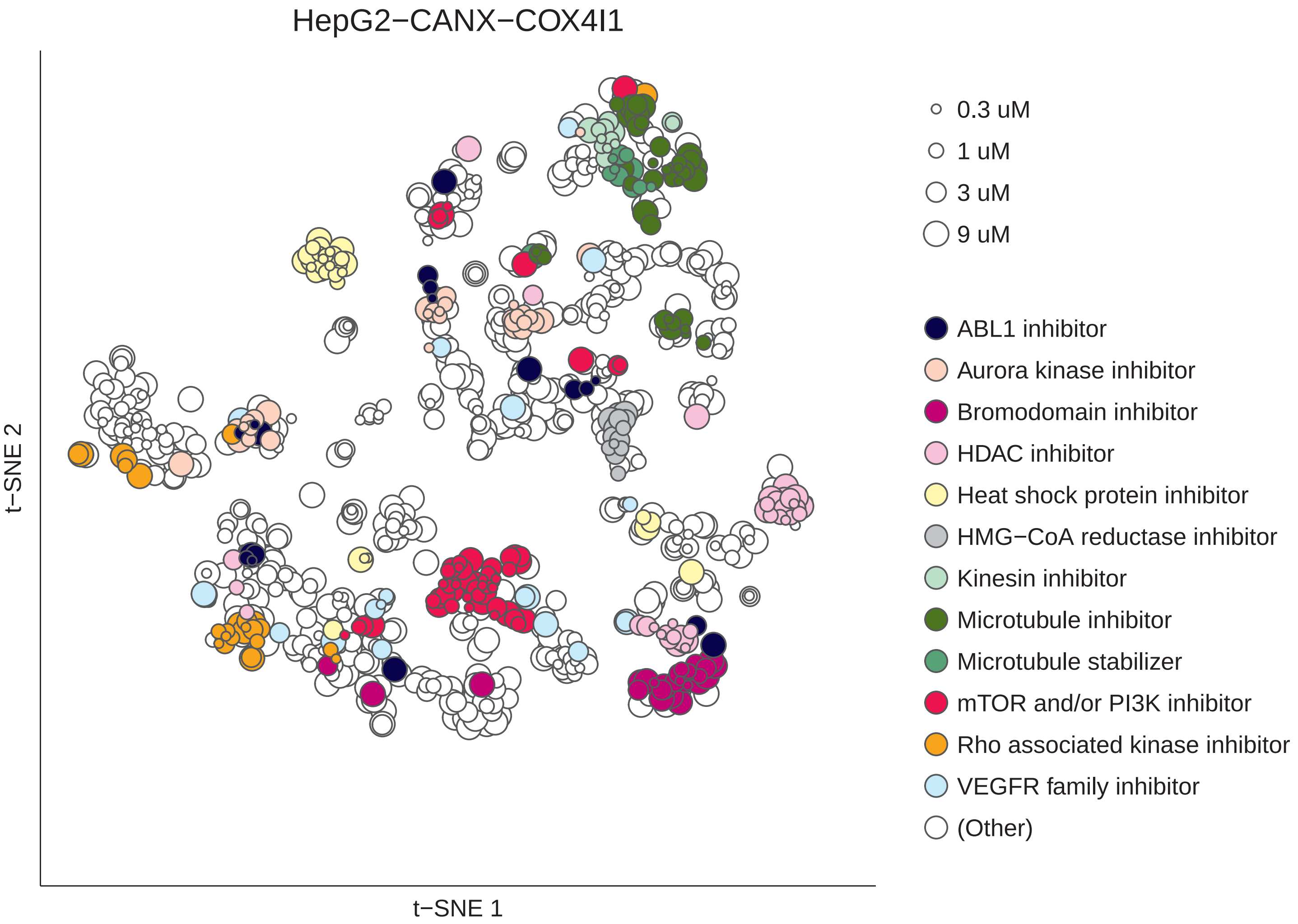
HepG2−CANX−COX4I1
t−SNE 2
t−SNE 1
0.3 uM
1 uM
3 uM
9 uM
ABL1 inhibitor
Aurora kinase inhibitor
Bromodomain inhibitor
HDAC inhibitor
Heat shock protein inhibitor
HMG−CoA reductase inhibitor
Kinesin inhibitor
Microtubule inhibitor
Microtubule stabilizer
mTOR and/or PI3K inhibitor
Rho associated kinase inhibitor
VEGFR family inhibitor
(Other)

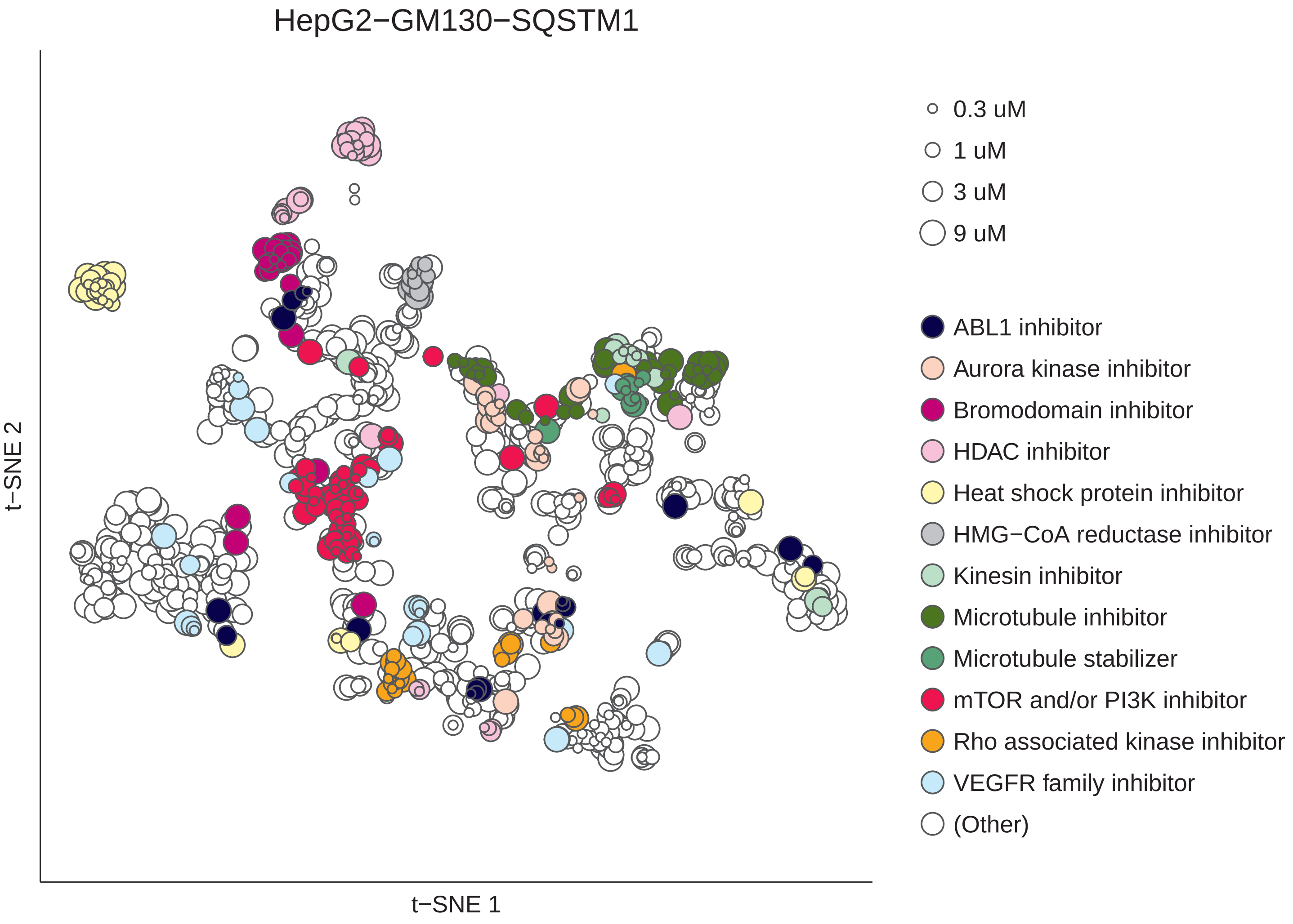

HepG2−GM130−SQSTM1
t−SNE 2
t−SNE 1
0.3 uM
1 uM
3 uM
9 uM
ABL1 inhibitor
Aurora kinase inhibitor
Bromodomain inhibitor
HDAC inhibitor
Heat shock protein inhibitor
HMG−CoA reductase inhibitor
Kinesin inhibitor
Microtubule inhibitor
Microtubule stabilizer
mTOR and/or PI3K inhibitor
Rho associated kinase inhibitor
VEGFR family inhibitor
(Other)

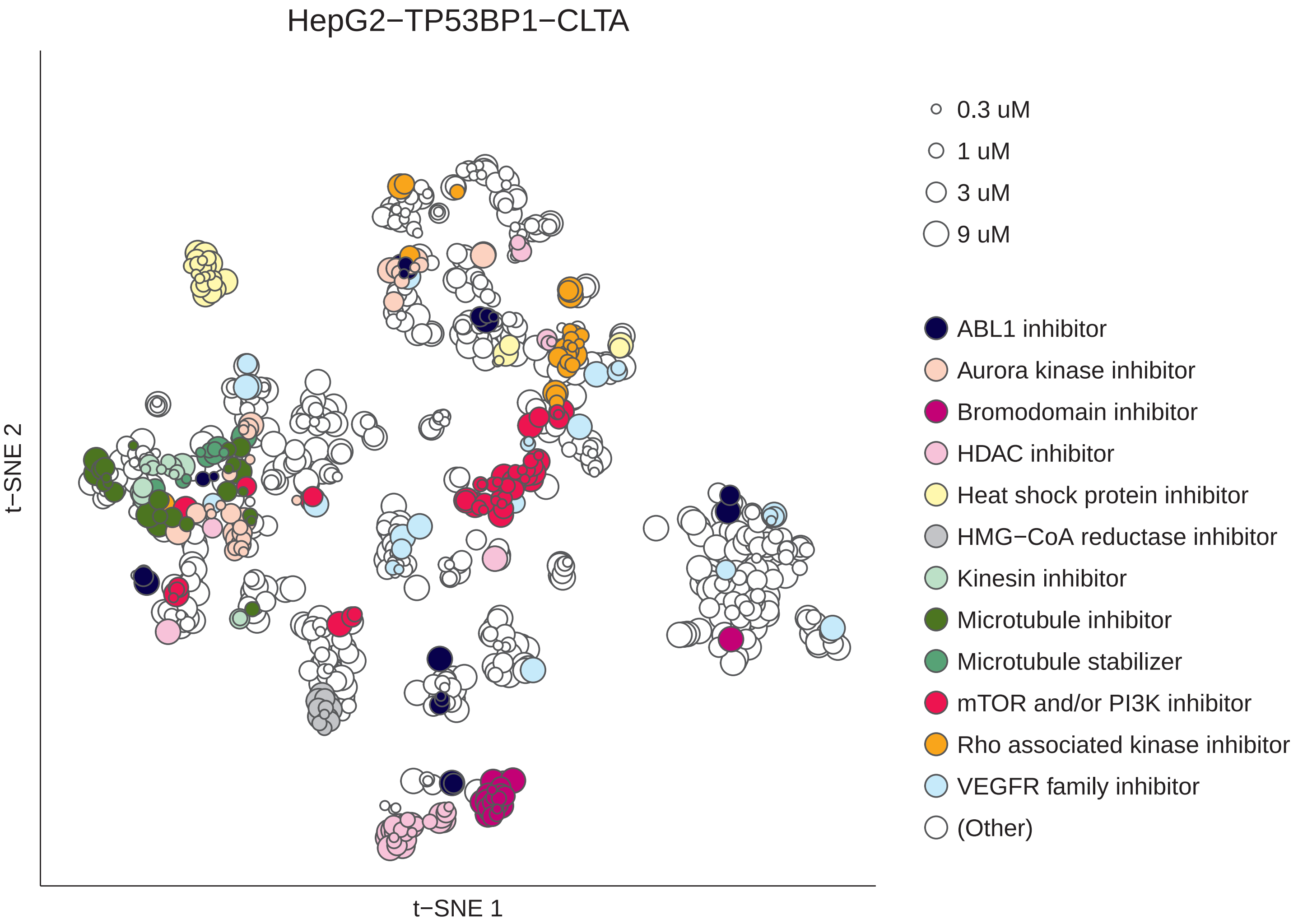
HepG2−TP53BP1−CLTA
t−SNE 2
t−SNE 1
0.3 uM
1 uM
3 uM
9 uM
ABL1 inhibitor
Aurora kinase inhibitor
Bromodomain inhibitor
HDAC inhibitor
Heat shock protein inhibitor
HMG−CoA reductase inhibitor
Kinesin inhibitor
Microtubule inhibitor
Microtubule stabilizer
mTOR and/or PI3K inhibitor
Rho associated kinase inhibitor
VEGFR family inhibitor
(Other)

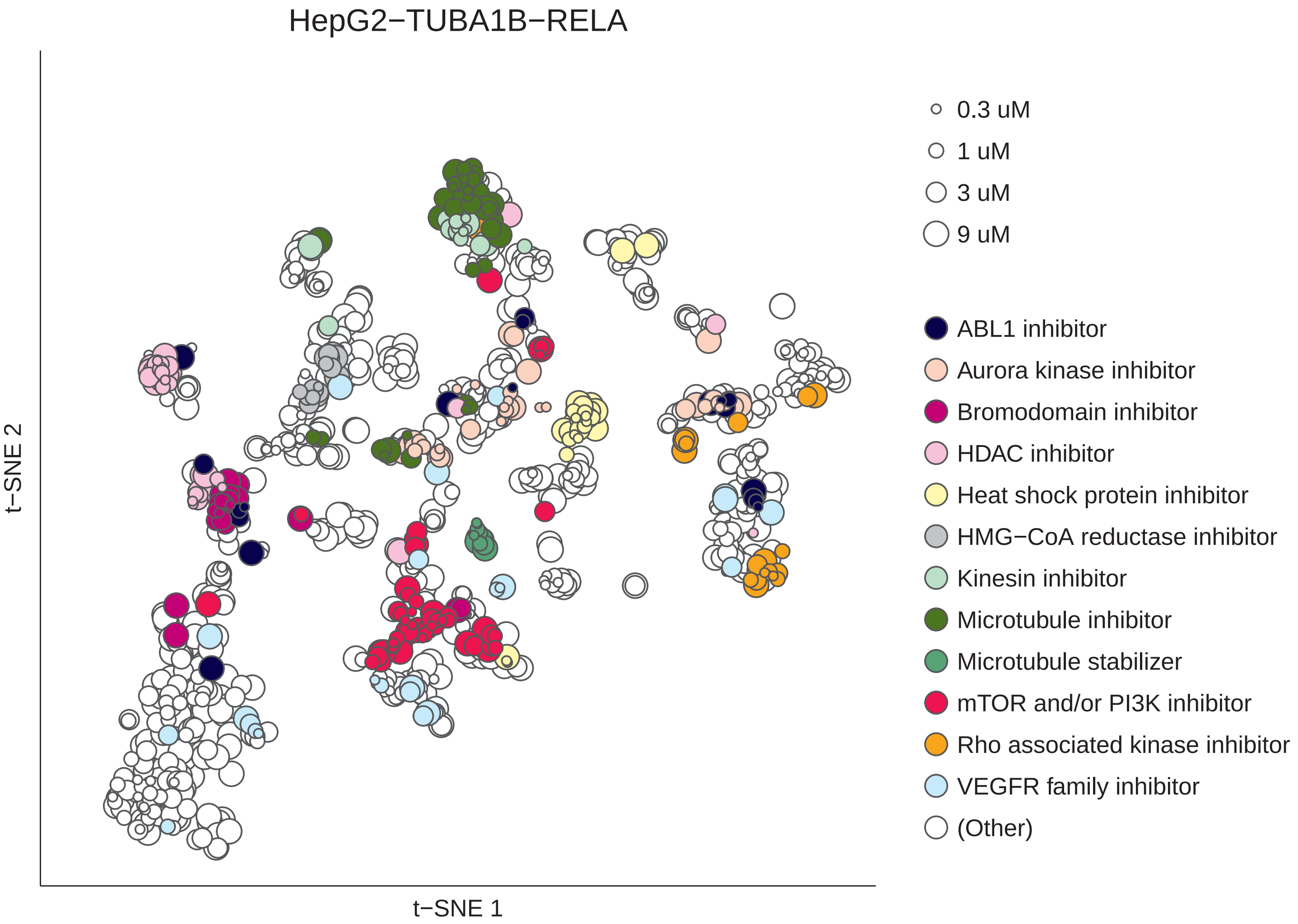
HepG2−TUBA1B−RELA
t−SNE 2
t−SNE 1
0.3 uM
1 uM
3 uM
9 uM
ABL1 inhibitor
Aurora kinase inhibitor
Bromodomain inhibitor
HDAC inhibitor
Heat shock protein inhibitor
HMG−CoA reductase inhibitor
Kinesin inhibitor
Microtubule inhibitor
Microtubule stabilizer
mTOR and/or PI3K inhibitor
Rho associated kinase inhibitor
VEGFR family inhibitor
(Other)

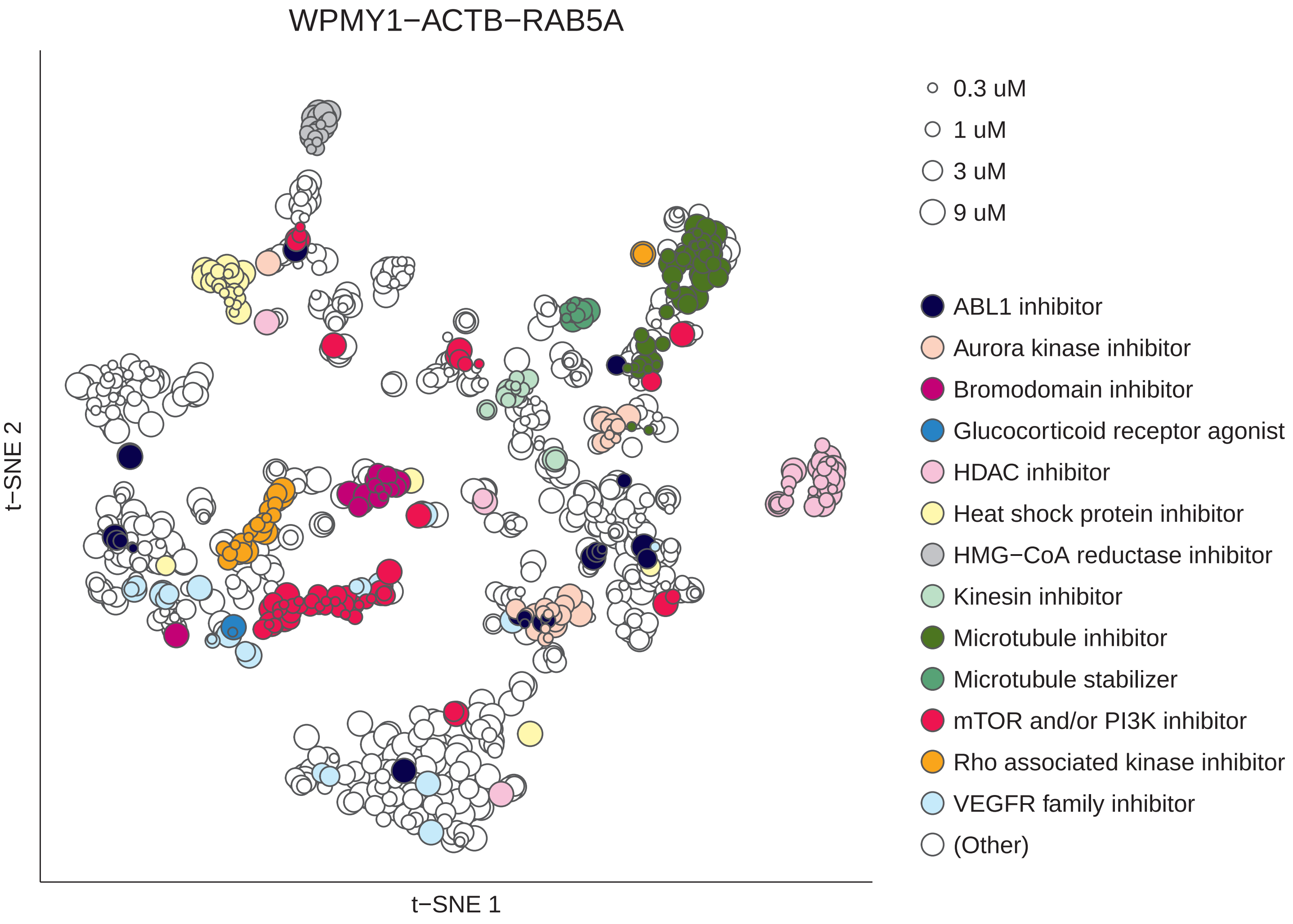
WPMY1−ACTB−RAB5A
t−SNE 2
t−SNE 1
0.3 uM
1 uM
3 uM
9 uM
ABL1 inhibitor
Aurora kinase inhibitor
Bromodomain inhibitor
Glucocorticoid receptor agonist
HDAC inhibitor
Heat shock protein inhibitor
HMG−CoA reductase inhibitor
Kinesin inhibitor
Microtubule inhibitor
Microtubule stabilizer
mTOR and/or PI3K inhibitor
Rho associated kinase inhibitor
VEGFR family inhibitor
(Other)

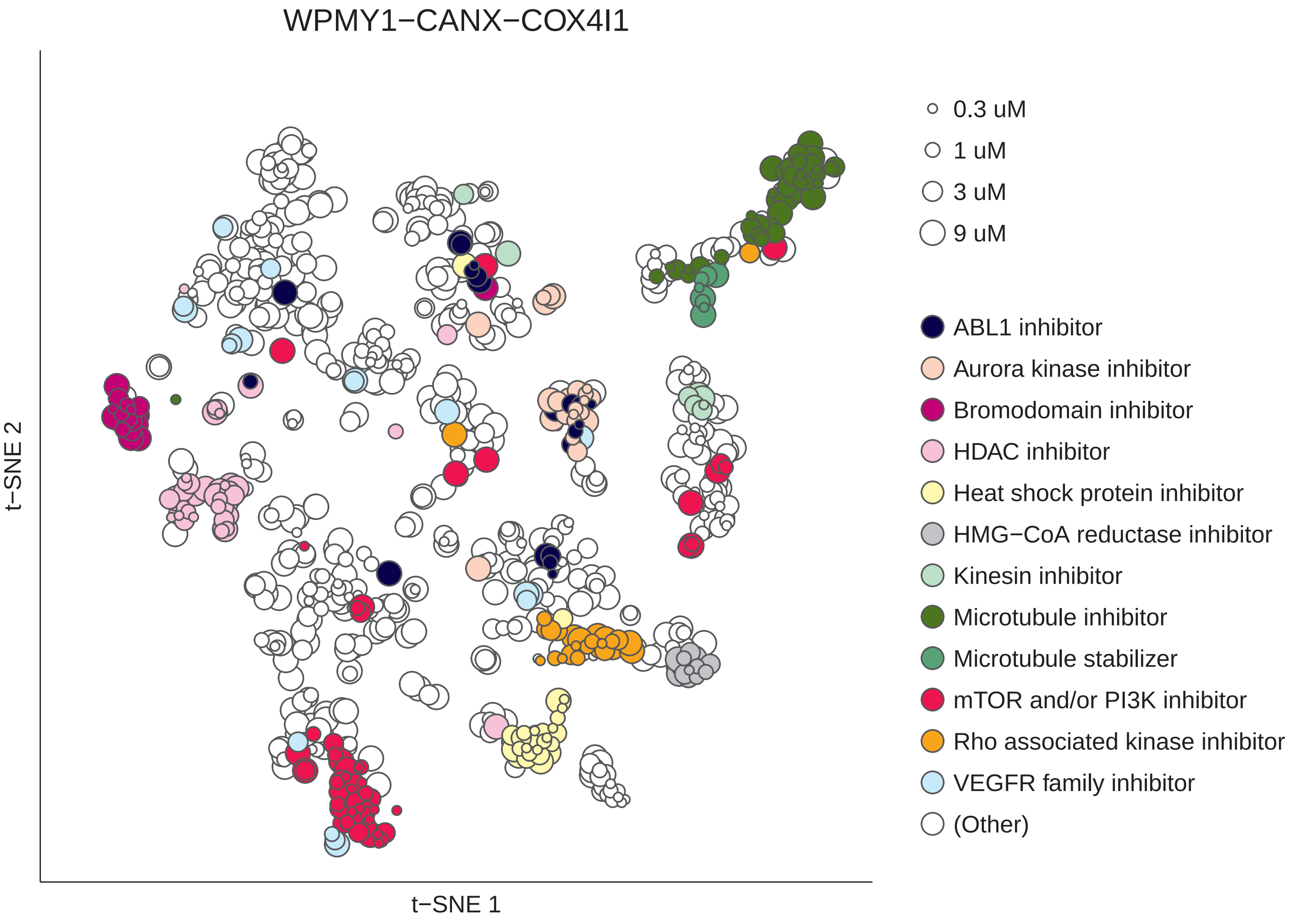
WPMY1−CANX−COX4I1
t−SNE 2
t−SNE 1
0.3 uM
1 uM
3 uM
9 uM
ABL1 inhibitor
Aurora kinase inhibitor
Bromodomain inhibitor
HDAC inhibitor
Heat shock protein inhibitor
HMG−CoA reductase inhibitor
Kinesin inhibitor
Microtubule inhibitor
Microtubule stabilizer
mTOR and/or PI3K inhibitor
Rho associated kinase inhibitor
VEGFR family inhibitor
(Other)

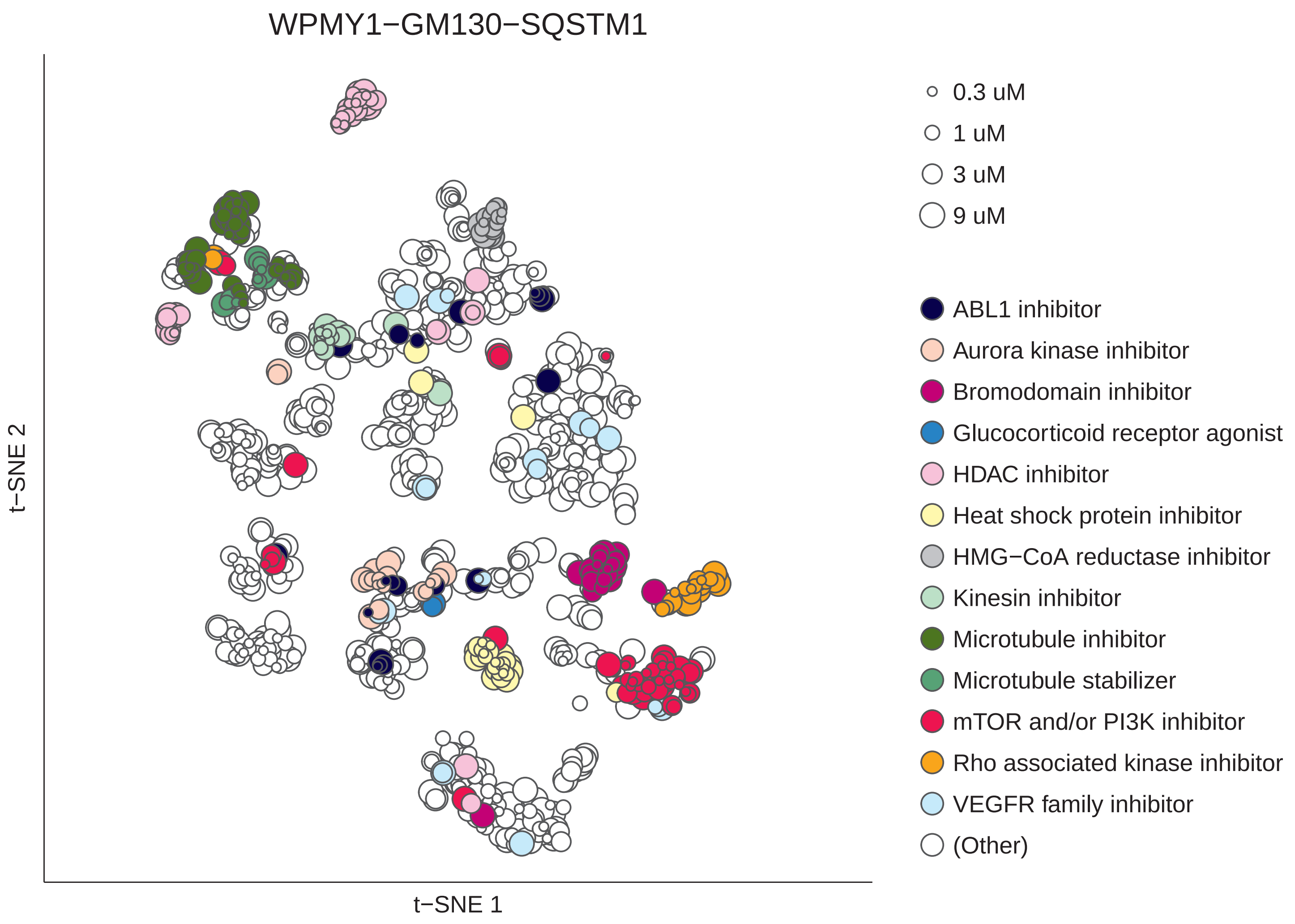

WPMY1−GM130−SQSTM1
t−SNE 2
t−SNE 1
0.3 uM
1 uM
3 uM
9 uM
ABL1 inhibitor
Aurora kinase inhibitor
Bromodomain inhibitor
Glucocorticoid receptor agonist
HDAC inhibitor
Heat shock protein inhibitor
HMG−CoA reductase inhibitor
Kinesin inhibitor
Microtubule inhibitor
Microtubule stabilizer
mTOR and/or PI3K inhibitor
Rho associated kinase inhibitor
VEGFR family inhibitor
(Other)

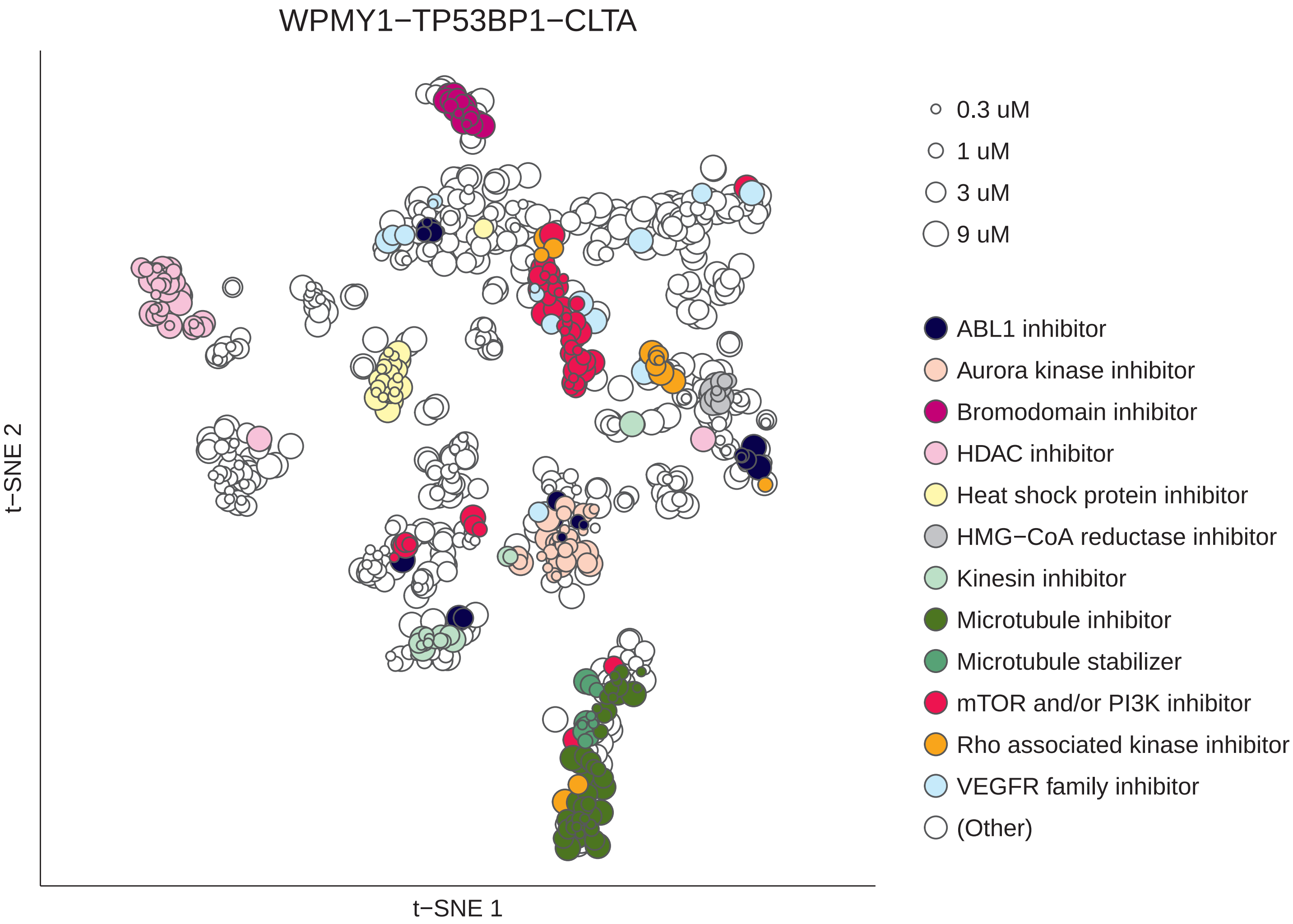
WPMY1−TP53BP1−CLTA
t−SNE 2
t−SNE 1
0.3 uM
1 uM
3 uM
9 uM
ABL1 inhibitor
Aurora kinase inhibitor
Bromodomain inhibitor
HDAC inhibitor
Heat shock protein inhibitor
HMG−CoA reductase inhibitor
Kinesin inhibitor
Microtubule inhibitor
Microtubule stabilizer
mTOR and/or PI3K inhibitor
Rho associated kinase inhibitor
VEGFR family inhibitor
(Other)

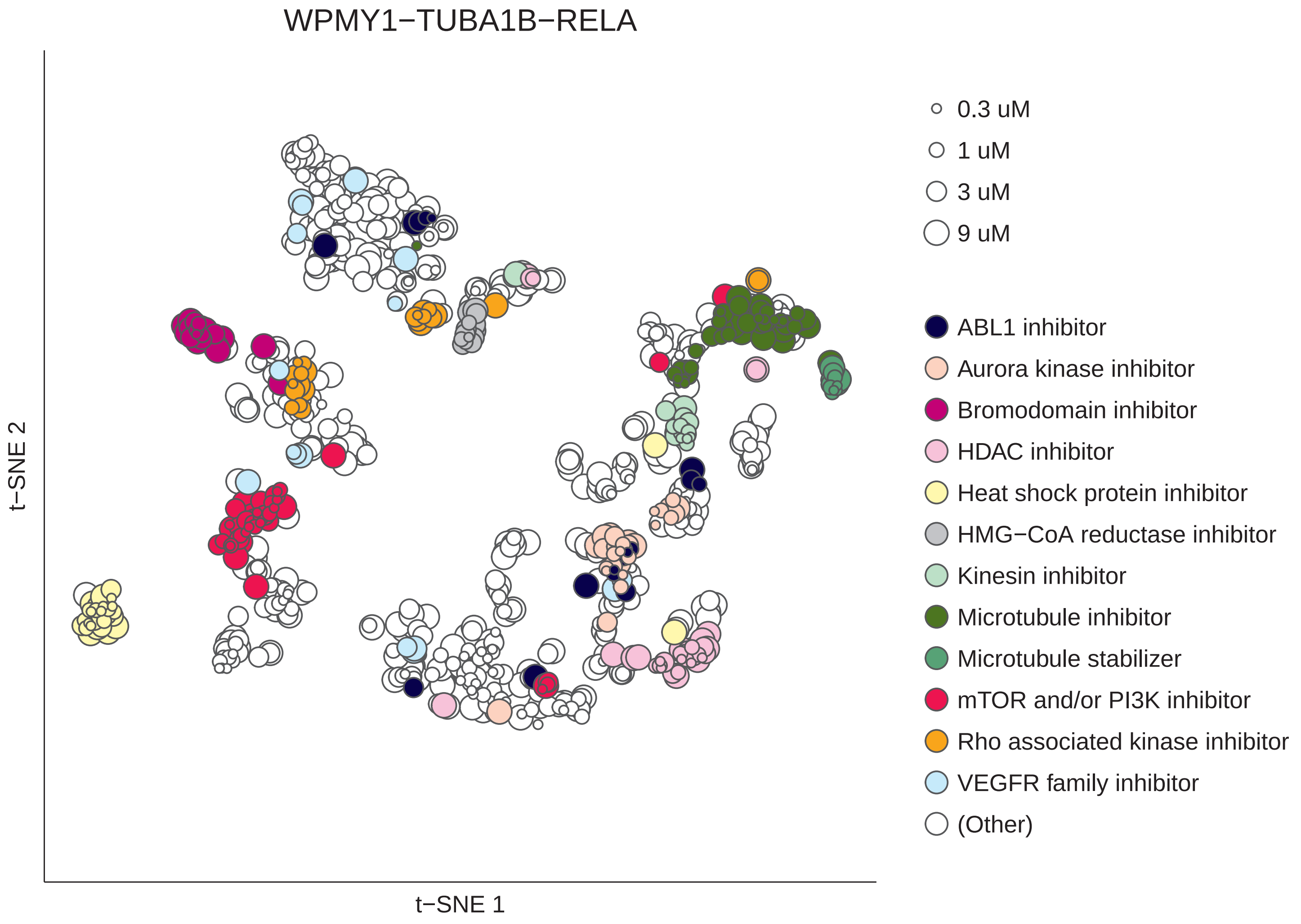
WPMY1−TUBA1B−RELA
t−SNE 2
t−SNE 1
0.3 uM
1 uM
3 uM
9 uM
ABL1 inhibitor
Aurora kinase inhibitor
Bromodomain inhibitor
HDAC inhibitor
Heat shock protein inhibitor
HMG−CoA reductase inhibitor
Kinesin inhibitor
Microtubule inhibitor
Microtubule stabilizer
mTOR and/or PI3K inhibitor
Rho associated kinase inhibitor
VEGFR family inhibitor
(Other)
